# Supplementary material for: SAMCell: Generalized label-free biological cell segmentation with segment anything
Source: PLoS One. 2025 Sep 8;20(9):e0319532. doi: 10.1371/journal.pone.0319532 (PMC12416835; doi:10.1371/journal.pone.0319532)
Supplement: S1 Appendix — Extended experimental details and results for training dataset configurations, model variant comparison (SAM-Base vs SAM-Large), patch size analysis, pretraining vs random initialization, post-processing thresholds, and training progression. (PDF) [file pone.0319532.s001.pdf]

Ablation Studies

In this section, we present a series of ablation tests designed to systematically analyze the contribution of various model components and hyperparameters. By modifying specific aspects of our model, such as initialization, post-processing thresholds, patch size, and training data configuration, we can quantify their individual effects on performance. In particular, we primarily evaluate these changes on our datasets, PBL-N2a and PBL-HEK, to rigorously assess the model's ability to generalize to previously unseen data. We also elect to use LIVECell and Cellpose Cytoplasm datasets for the pretraining study to better highlight the effect of pretraining. These experiments provide critical insights into the strengths and limitations of our approach, guiding future improvements.

Training Dataset

Examining the performance of different training dataset configurations reveals interesting patterns. For the PBL-N2a dataset, SAMCell trained on the Cellpose Cytoplasm dataset (SAMCell-Cyto) outperforms SAMCell trained on the LIVECell dataset (SAMCell-LiveCell). Conversely, for the PBL-HEK dataset, SAMCell-LiveCell slightly outperforms SAMCell-Cyto. This suggests that each training dataset contributes unique information relevant to different cell morphologies and imaging conditions.

The Cellpose Cytoplasm dataset, despite its smaller size (approximately 500 images compared to around 3000 in LIVECell), likely provides better generalization for cells with circular morphology like N2a due to its diverse collection assembled via internet scraping. This process results in images derived from a wide variety of cell lines and imaging techniques. Meanwhile, the LIVECell dataset's more homogeneous collection of eight cell lines imaged via phase contrast appears to better capture the characteristics of densely packed HEK cells.

	SAMCell-Generalist (Cyto+LIVECell)	SAMCell-Cyto (Cytoplasm only)	SAMCell-LiveCell (LIVECell only)
	Test Dataset: *PBL-HEK*		
SEG	**0.425140**	0.295300	0.298700
DET	**0.771509**	0.613500	0.634500
OP_{CSB}	**0.598325**	0.454400	0.466600
	Test Dataset: *PBL-N2a*		
SEG	**0.706734**	0.696400	0.569500
DET	**0.941132**	0.918400	0.871100
OP_{CSB}	**0.823933**	0.807400	0.720300

Table 1. Performance comparison between SAMCell fine-tuned on different training dataset combinations. The parentheses contain the names of the training datasets for each configuration.

The highest values for SEG, DET, and OP_{CSB} are achieved when training with a combination of the Cellpose Cytoplasm and LIVECell datasets for SAMCell-Generalist, as shown in Table 1. This combined model consistently outperforms both individual models across all metrics on both datasets. The synergistic effect of this combined training strategy can be attributed to three main factors: 1) increased sample size, which enhances the robustness of the learned representations, 2) greater diversity of cell morphologies and imaging conditions, which provides complementary information that improves the model's ability to generalize, and 3) the ability to learn dataset-specific strengths from each source. By exposing SAMCell to a broader spectrum of cell types during training, the model develops more versatile representations that can better

handle variations in cell density, contrast, and morphology, leading to superior segmentation and detection performance.

These results emphasize the importance of dataset diversity and complementarity in training deep learning models for biomedical image segmentation, suggesting that merging datasets with different strengths can significantly boost performance in zero-shot, cross-dataset scenarios where the test data may differ considerably from training examples.

Model Variant: SAM-Base vs SAM-Large

Having understood that concatenating datasets produces optimal results, we conducted an ablation study with this generalist strategy to evaluate the impact of using different SAM model variants as the foundation for our SAMCell approach. Specifically, we compared the performance of SAMCell based on SAM-Base (ViT-B) versus SAM-Large (ViT-L) on our zero-shot datasets. As shown in Table 2, the SAM-Large variant consistently outperforms the SAM-Base variant across all metrics and datasets.

	SAMCell Generalist SAM-L	SAMCell Generalist SAM-B
	Test Dataset: *PBL-HEK*	
SEG	**0.471126**	0.425140
DET	**0.803439**	0.771509
OP_{CSB}	**0.637282**	0.598325
	Test Dataset: *PBL-N2a*	
SEG	**0.741098**	0.706734
DET	**0.942240**	0.941132
OP_{CSB}	**0.841669**	0.823933

Table 2. Performance comparison between SAM-Large and SAM-Base model variants.

These results suggest that the larger capacity of the SAM-Large model allows for better feature extraction and generalization to unseen cell types and imaging conditions. However, this improvement comes with increased computational requirements and inference time. For our main experiments, we used the SAM-Base variant to balance performance and computational efficiency, particularly for the user-facing application where processing speed is important.

Patch Size

After examining the impact of model size, we next investigate the optimal patch size for processing images. The Segment Anything Model's output size is 256×256, which means that if we want to use Meta's pretrained weights and not change the model architecture, we must work with this. This means that when we have a patch size different from 256, we must interpolate the outputs. This observation led to the hypothesis that using a patch size of 256 would yield the highest segmentation accuracy since it avoids any resampling artifacts.

	SAMCell-Generalist		
	128	256	512
	Test Dataset: *PBL-HEK*		
SEG	0.330196	**0.425140**	0.347062
DET	0.644326	**0.771509**	0.733211
OP_{CSB}	0.487261	**0.598325**	0.540137
	Test Dataset: *PBL-N2a*		
SEG	**0.712682**	0.706734	0.598327
DET	0.926788	**0.941132**	0.931232
OP_{CSB}	0.819735	**0.823933**	0.764780

Table 3. Performance Comparison between using different patch sizes.

Table 3 reports the performance metrics for three different patch sizes evaluated on the PBL-HEK and PBL-N2a datasets. This study was done using SAMCell-Generalist (SAM-B trained on both the Cellpose Cytoplasm and LIVECell datasets). These findings confirm our hypothesis: aligning the patch size with the native output

dimensions of the model minimizes interpolation errors and yields superior segmentation and detection results. Consequently, a patch size of 256 is the preferred setting for our experiments to ensure optimal accuracy.

Pretraining

We now explore the critical question of whether SAM's extensive pretraining contributes meaningfully to performance in cell segmentation tasks. Approaches based on the Segment Anything Model naturally inherit pretrained weights from the extensive dataset used to train SAM. To gauge pretraining's impact, we conduct an ablation study comparing SAMCell as described, finetuned starting from Meta's pretrained weights [**?**], to a SAMCell variant trained starting from random weights. Test set performance on both LIVECell and Cellpose Cytoplasm datasets is shown in Table 4. The significant drop in performance when random weight initialization is used instead of pretrained weights suggests pretraining has a strong impact on SAMCell's performance.

To further explore the impact of pretraining on generalization capabilities, we extend our investigation to zero-shot, cross-dataset performance. Table 4 shows the performance of SAMCell-Generalist with pretrained and random initialization on our two evaluation datasets. Similar to the test set results, we observe a dramatic drop in performance across all metrics when using random initialization, reinforcing the importance of pretraining for cross-dataset generalization.

	Test-set Performance		Zero-Shot Performance	
	SAMCell	SAMCell-rand	SAMCell Generalist	SAMCell-rand Generalist
	Train & Test Dataset: *Livecell*		Test Dataset: *PBL-HEK*	
SEG	**0.651876**	0.106720	**0.425140**	0.228275
DET	**0.892543**	0.510541	**0.771509**	0.607700
OP_{CSB}	**0.772210**	0.308631	**0.598325**	0.417988
	Train & Test Dataset: *Cellpose Cytoplasm*		Test Dataset: *PBL-N2a*	
SEG	**0.611144**	0.132846	**0.706734**	0.579669
DET	**0.865908**	0.430565	**0.941132**	0.874621
OP_{CSB}	**0.738526**	0.281701	**0.823933**	0.727145

Table 4. Performance comparison between SAMCell using pretrained weights and random initialization: test-set performance (left) and zero-shot, cross-dataset performance (right).

The training progression for both initialization approaches, shown in Fig 1, reveals important insights into the value of pretraining. The pretrained model demonstrates strong performance as early as epoch 5, with only modest improvements over subsequent epochs. This suggests that the rich features learned during SAM's extensive pretraining provide an excellent foundation that requires minimal fine-tuning to adapt to cell segmentation tasks. The model with random initialization, while showing continuous improvement throughout training, consistently underperforms compared to the pretrained model, even after many more epochs of training.

These results highlight two key advantages of leveraging pretrained weights: 1) superior performance even after full convergence, and 2) the potential for extremely efficient fine-tuning with as few as 5 epochs for practical applications where computational resources are limited. The substantial performance gap between pretrained and randomly initialized models across all datasets confirms that SAM's pretraining on diverse natural images provides transferable representation learning benefits for specialized tasks like cell segmentation.

Fig 1. Training progress for pretrained and random weights for (a) PBL-HEK and (b) PBL-N2a, highlighting how early stopping stops the training once the model converges.

Post-Processing Thresholds

With the model architecture and training strategy optimized, we turn our attention to the post-processing pipeline, which is crucial for converting the predicted distance maps into discrete cell masks. As described in Section **??**, post-processing is required to convert the predicted distance map into a set of discrete cell masks. This process involves thresholding the continuous-valued output of the model to generate two binary masks. The first mask, which we refer to as the *binary mask*, indicates whether each pixel belongs to a cell or to the background. This mask is obtained by applying a threshold value, denoted as cell fill threshold, such that all pixels with distance map values greater than this threshold are considered part of a cell.

Fig 2. Threshold parameter space visualization for (a) PBL-HEK and (b) PBL-N2a.

The second mask, referred to as the *cell centers* mask, is used to identify seed points for the watershed algorithm. This mask is expected to contain exactly one connected component within each cell's body. It is computed using a higher threshold value, denoted as cell peak threshold, applied in the same manner.

To determine the optimal values for these post-processing thresholds, we conduct an ablation study across a range of cell fill threshold and cell peak threshold values. Specifically, we evaluate the performance of SAMCell-Generalist (SAM-B trained on both the Cellpose Cytoplasm and LIVECell datasets) with a patch size of 256. For each pair of threshold values, we compute the corresponding $\mathrm{OP}_{\mathrm{CSB}}$ score to assess overall segmentation quality.

The results are visualized in Fig 2, which illustrates the impact of threshold choices on performance across the datasets. Across datasets, we observe that the optimal cell peak threshold and cell fill threshold values differ slightly, reflecting dataset-specific characteristics such as cell density, morphology, and contrast. However, when considering generalization across all datasets, we find that a global setting of cell peak threshold = 0.47 and cell fill threshold = 0.09 yields the best average performance, achieving a mean $\mathrm{OP}_{\mathrm{CSB}}$ score of 0.715077.
