# Supplementary figures and images for: SAMCell: Generalized label-free biological cell segmentation with segment anything
Source: PLoS One. 2025 Sep 8;20(9):e0319532. doi: 10.1371/journal.pone.0319532 (PMC12416835; doi:10.1371/journal.pone.0319532)

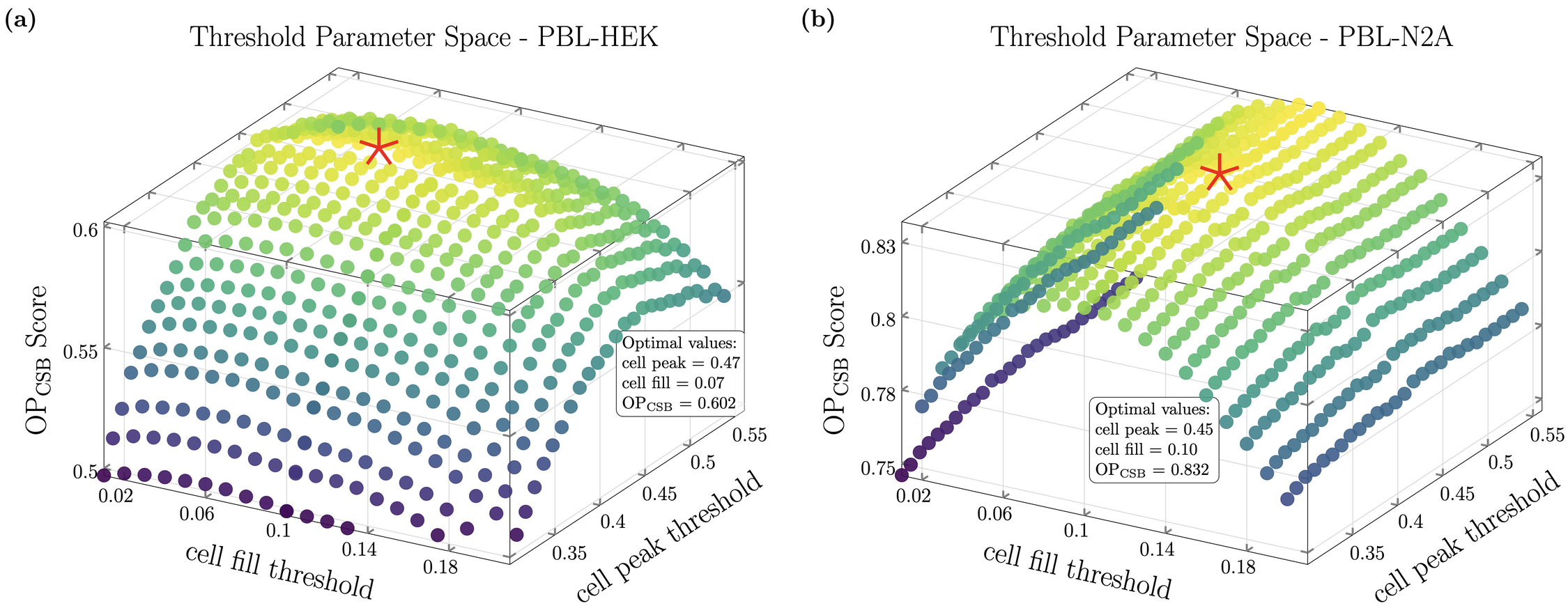

Supplement: S1 File — (ZIP) [file pone.0319532.s002.zip › fig2.tif]

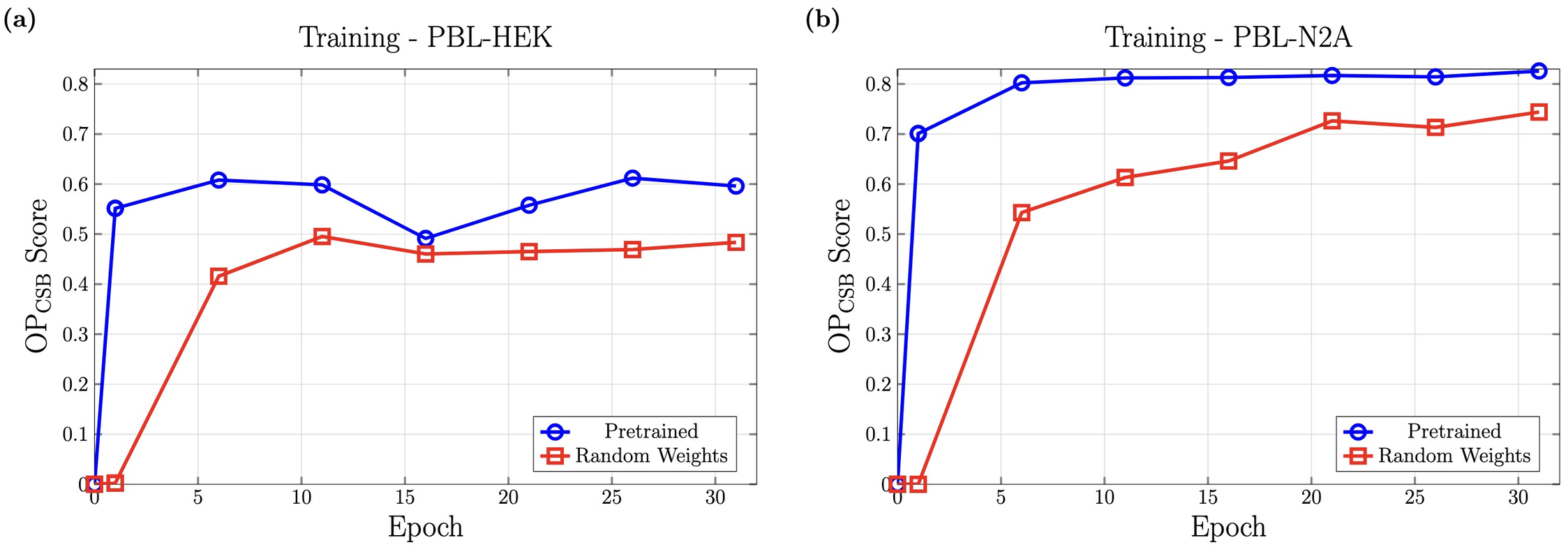

Supplement: S1 File — (ZIP) [file pone.0319532.s002.zip › fig1.tif]
