# Supplementary material for: SAMCell: Generalized label-free biological cell segmentation with segment anything
Source: PLoS One. 2025 Sep 8;20(9):e0319532. doi: 10.1371/journal.pone.0319532 (PMC12416835; doi:10.1371/journal.pone.0319532)
Supplement: Attachment — Submitted filename: Response to Reviewers SamCell Rev2.pdf [file pone.0319532.s004.pdf]

We thank the reviewer for their remaining feedback and diligence in reading our manuscript. We have read and carefully addressed each point raised, both below and in the text.
Thank you also to everyone involved in this process, including the other reviewers who did not have any requested revisions at this stage and the editor. We look forward to completing the peer review process and taking this manuscript across the finish line!

1. One-sentence paragraphs are present; consider merging or expanding these for better readability and flow.

Thanks for this note. We have merged any unintended one sentence paragraphs.

2. There are few figures with the same label.

Thanks for this comment. We are not sure which figures this refers to, as all of the figures have the correct label in our copy of the manuscript. It is possible that the compiling of the paper has caused you to see something different than we do. We will check with the editor to ensure that there is no mislabeling when the paper is published.

3. Please specify the imaging modality for each dataset used in the paper — for instance, whether the data comes from fluorescence microscopy, IHC staining, or another technique. Possibly, report results on other modalities than phase contrast and light microscopy, or specifically mention that the methodology is suitable for those types of microscopic images.

We appreciate the reminder. In this paper, our methodology is specifically optimized for phase-contrast and bright-field microscopy images, which represent the majority of label-free cell imaging applications and is the focus of our paper as a whole. We absolutely would consider publishing a paper that specifically considers fluorescently labeled datasets in the future.

We have ensured that the imaging modality is included for all the datasets mentioned in the paper. In summary, in the paper the following imaging modalities are specified:

- *LIVECell: "phase-contrast images"*
- *Cytoplasm: "various microscopy techniques, including both bright-field and fluorescent images"*
- *PBL-HEK and PBL-N2a: "phase-contrast microscopy images"*

4. In the DET evaluation, the term “empty graph” should be clarified.

Thanks for this request for clarification. We now state that "empty graph" represents a graph with no nodes (no detected cells) by adding the following to Section 2.2: "...an empty graph (a graph with no nodes, representing no detected cells) into…"

5. Additionally, define AOGM-D explicitly so that readers unfamiliar with this metric can follow the evaluation logic.

Thank you for the suggestion. We added an explicit definition of AOGM-D as quantifying the minimum cost of transforming one graph representation into another, by rephrasing the paragraph in Section 2.2 as follows:

"This is done with a graph-based approach, the "Acyclic Oriented Graph Matching Measure for Detection" (AOGM-D), which quantifies the minimum cost of transforming one graph representation into another through elementary graph operations. To compute the AOGM-D, we first generate graphs from the ground truth and model prediction, with each cell serving as a node. Then, the number of basic graph operations (e.g., add node, remove node, split node into two) required to convert the predicted graph into the ground truth graph is computed.}"

6. The authors claim the MedSAM and SAMed models do not work well. But, they did not provide any evidence of this claim.

Thanks for this comment, we will try to clarify. MedSAM and SAMed were not designed for cell segmentation—MedSAM targets medical imaging modalities like CT scans and X-rays, while SAMed focuses on organ segmentation in CT images. Therefore, directly applying these methods to cell segmentation would not be appropriate, and rationally would not be expected to perform even to the level of Default SAM(results shown in Figure 5). We refer to these works solely to present context for different fine-tuning strategies and architectural choices for adapting SAM to specialized domains.

More specifically,

- *MedSAM-style approaches would be impractical due to requiring bounding box prompts for hundreds of cells per image*
- *SAMed-style categorical approaches cannot distinguish individual cell instances when cells are touching—a critical requirement for cell segmentation*

We've enhanced the manuscript to clearly distinguish between the original applications of these methods and the theoretical limitations their strategies would face if adapted to cell segmentation. Our preliminary experiments with categorical segmentation

approaches (inspired by SAMed's strategy) confirmed these theoretical limitations. We acknowledge that a comprehensive study of potential modifications to make these approaches suitable for cell segmentation could be valuable future work, but is beyond the scope of this study.

Added/Modified in Section 1:

“It is important to note that neither MedSAM nor SAMed were designed for cell segmentation, and therefore directly applying these methods to cell segmentation would not be appropriate or meaningful. However, both works give important insight into successful fine-tuning strategies and architectural choices for adapting SAM in a specialized domain. Inspired by MedSAM's encouraging segmentation performance of organs, we take a similar approach of fine-tuning the SAM image encoder for our particular dataset. However, we expand upon their work by eliminating the need for a bounding box prompt, which would be impractical for cell segmentation where hundreds of cells may be present in a single image. Similarly, while SAMed demonstrates semantic segmentation without prompts, we extend this approach by conducting unprompted instance segmentation rather than being limited to discrete categories, which cannot distinguish individual cell instances when cells are touching or overlapping.”

7. The authors mentioned problems in distance transform of peanut and C-shaped cells. They did not address the problem further. Are they removed from the training set, or are they the ones lowering the SEG and DET scores?

Thanks for your question. We expanded the discussion to clarify these are edge cases that may contribute to lower scores and were not removed from our realistic datasets.

Updated text in Section 2.4:

“This approach does have some drawbacks for cells consisting of multiple compartments. Namely, when a cell is peanut-shaped, like those formed when a cell is in the process of splitting, SamCell would consistently segment this shape as two cells, while an expert annotator may segment it either as one cell or two cells, depending on the person. While peanut-shape morphologies are a minority under typical cell culture conditions, we suspect that they may thus contribute to lower SEG and DET scores. For other non-circular shapes (like ovoid or "C-shaped" cells) we observe through empirical testing (data not shown) that the postprocessing technique can properly recover their geometries. It is important to note that our training and testing datasets

contain realistic cell morphologies and expert annotations, without removing any problematic cases, ensuring our evaluation reflects real-world performance, so edge-cases like peanut-shaped cells are not removed from the dataset.”

8. In the second paragraph of Section 2.5.2., why is there a need to upsample already large image?

Good question! Thanks for highlighting this so we have the chance to address it. The primary reason is that SAM's architecture takes 1024×1024 inputs and produces 256×256 outputs. By using 256×256 patches, we can directly compare the original patch with the model's output, maintaining pixel-level correspondence for training and evaluation. Additionally, microscopy images are often much larger than SAM's required 1024×1024 input (e.g., 2048×2048 or larger). Direct resizing would cause distortions and loss of cellular detail through downsampling for large images. Our approach preserves original resolution within each 256×256 patch by consistently upsampling discrete regions to 1024×1024, maintaining the cellular features essential for accurate segmentation. We have clarified this in the text by adding/modifying the following in Section 2.5.2:

"We chose this sliding window approach over directly resizing the entire input image to 1024 x 1024 for two reasons. First, SAM's architecture takes 1024 x 1024 inputs and produces 256 x 256 outputs. By using 256 x 256 patches, we can directly compare the original patch with the model's output without further manipulation, maintaining pixel-level correspondence for training and evaluation. Second, microscopy images often contain thousands of pixels in each dimension (e.g., 2048 x 2048 or larger). Direct resizing of such large images to SAM's required 1024 x 1024 input could result in a catastrophic distortion or loss of cellular detail. Our sliding window approach preserves the original resolution by consistently upsampling 256 x 256 regions to 1024 x 1024, an approach which maintains the cellular features essential for accurate segmentation."

9. In Section 2.5.5, it is confusing whether the watershed algorithm starts from the center of the cell or from the background. Authors mention that watershed starts from local minima.

Thanks for this question. The algorithm treats our distance map as a topographical surface where cell centers are peaks and boundaries are valleys, starting from seed points(in the center of the cell) and then determining boundaries where flood regions from different seeds meet. We have clarified the watershed algorithm explanation to better describe how it operates in our specific context.

Added/Modified in Section 2.5.5:

"We apply the Watershed algorithm, a classical approach to boundary detection, to recover cell masks from the distance map. The algorithm treats the distance map as a topographical surface where cell centers represent peaks and cell boundaries represent valleys. Starting from the cell centers identified by the cell peak threshold, the algorithm simulates water flooding toward the simulated valleys. The cell boundaries are thus determined where the flood regions hit a clear boundary (the cell edge), or when the flood regions from different cell centers meet (if a cell edge is not clear), effectively separating individual cells even when they are densely packed…."

10. The authors refer to MedSAM and SAMed, but does show the results. Results from those models should be included in the evaluation, or at least show evidence that they do not segment correctly.

Thank you for this question, as your point is similar to that of Question 6, (The authors claim the MedSAM and SAMed models do not work well. But, they did not provide any evidence of this claim.) for brevity, we have addressed this point in our response to your question 6.
