# Supplementary material for: SAMCell: Generalized label-free biological cell segmentation with segment anything
Source: PLoS One. 2025 Sep 8;20(9):e0319532. doi: 10.1371/journal.pone.0319532 (PMC12416835; doi:10.1371/journal.pone.0319532)
Supplement: Attachment — Submitted filename: Response to Reviewers SamCell.docx [file pone.0319532.s003.docx]

*April 21, 2025*

*Response to Reviewers*

*We thank the reviewers for their constructive feedback and valuable suggestions. Their comments have helped us improve the manuscript by making significant changes throughout and clarifying several important aspects of our work. In the marked up manuscript, additions to the text are colored in blue, and deletions are colored in red. Below, we address each point raised by the reviewers.*

**Reviewer #1: The paper introduces SAMCell, a modified version of Meta’s Segment Anything Model (SAM), designed for label-free biological cell segmentation. The authors aim to address the challenges of cell segmentation in microscopy images, such as vague cell boundaries and the transparent nature of cells. SAMCell is trained on large-scale datasets of microscopy images and demonstrates strong performance across various cell types and microscopy techniques. Additionally, the authors provide a user-friendly interface to make SAMCell accessible to biologists without machine learning expertise.**
**The experimental results demonstrate the algorithm's superiority. However, there are some weaknesses in this paper.**

**1. To enhance the paper, the authors should address the computational cost issue by exploring optimization techniques or lighter model variants that maintain performance while reducing resource requirements.**

*We appreciate this suggestion and have clarified our approach to managing computational costs in the manuscript. We have added this information to Section 2.5.4 Finetuning Methodology “To address computational efficiency…” In summary, after running an ablation study between SAM-Large and SAM-Base (Appendix A.2), we selected SAM-base, the smallest model variant, to minimize computational requirements while maintaining performance. We explored parameter-efficient fine-tuning approaches like LoRA but found they significantly reduced performance for cell segmentation tasks. Our current implementation delivers acceptable inference times, especially when using CUDA-enabled GPUs or cloud computing resources like Google Colab.* **2. Expanding the ablation study to include more comprehensive comparisons with random initialization would better highlight the value of pretraining.**

*Thank you for this suggestion. Upon further reflection, we agreed with the need for more precise ablation studies and actually upon performing them, we were able to optimize our parameters even further. This resulted in higher perfomance than previously seen, so we would like to humbly thank our reviewer for driving this change. We have included a comprehensive comparison between SAMCell with pretrained weights and with random initialization (SAMCell-rand) in our deeper ablation study in appendix A.4 which demonstrates the substantial performance gains from pretraining. We demonstrate performance on test and zero shot datasets, as well as the effect of pretraining on the ability of the model to converge. This comparison highlights the critical role that pretraining plays in our model's effectiveness. We have also conducted four other ablation studies, all in appendix A.*

**3. The authors should broaden the evaluation to include a wider range of microscopy techniques to demonstrate SAMCell’s versatility.**

*Thank you for the thoughtful suggestion. We would absolutely consider expanding our evaluation in future work. Unfortunately, due to the scarcity of properly annotated data sets, and the time it takes to annotate just one cell image, for this study we limited our test set creation to two scenarios. We trained on datasets which contain numerous cell types, microscope modes, and magnifications. For this research we sought to prove efficacy for our segmentation algorithm using very difficult images to segment: brightfield images from a low cost microscope. We have run other types of images through SAMCell, and anecdotally, the segmentations of, for example, fluorescent images appear much more accurate than brightfield images.*

**4. A detailed discussion of the post-processing steps, including the rationale behind the watershed algorithm choice and potential alternatives, would strengthen the methodology section.**

*Thank you for this thoughtful suggestion. We have expanded our discussion of the watershed algorithm in Section 2.5.5, detailing its rationale and implementation for converting distance maps to cell masks. We've also added information about potential alternatives to our approach, and the rationale behind why they weren’t chosen.*

**Reviewer #2: The paper modifies Segment Anything Model (SAM) for cell segmentation task of phase-contrast and brightfield microscopic images. In addition they publish their own dataset two cell lines. I like the idea of using distance maps as prediction task, and evaluation on the own datasets.**

**Here are the comments:**

**1. I do not agree with the claim saying, "...being based on U-Net, the model may under-fit larger datasets and has a reduced advantage from pretraining.". U-Net is an architecture where any large backbone network can be used. If underfitting is the problem, the straightforward solution would be to simply increase the network size, use a larger CNN, add more layers, etc.**

*Thank you for the correction, we have removed this sentence from section 1. Because U-Net is a small CNN, we thought it was important to point out the potential of underfitting, but it is now made clear in the text that the issue is not inherent to U-Net, but rather a general consequence of CNN size.*

**2. In my opinion, the number of parameters is never one of the main advantages. "...SAMCell inherits Segment Anything’s main advantages: a high parameter, ..."**

*Thank you for sharing this thought. Given that it is subjective whether number of parameters is an advantage, we have rephrased this sentence in section 1 from “"...SAMCell inherits Segment Anything’s main advantages: a high parameter, Vision Transformer based architecture” to “..SAMCell inherits Segment Anything’s main advantage: a Vision Transformer based architecture” instead to focus solely on the ViT based architecture.*

**3. In the Introduction section, contributions of the paper should be highlighted.**
**1. Prediction of distance-transform map**
**2.Creation and publication of novel dataset.**

*This is an excellent note, we have made the contributions of this paper clear in the introduction by adding the following sentences “Our main contributions are twofold: First, we introduce a novel approach that fine-tunes SAM to output a real-valued distance map describing the Euclidean distance to a cell border for each pixel in the image. We then recover the boundary using a post-processing technique based on the watershed algorithm to effectively address the challenging problem of segmenting densely packed cells with ambiguous boundaries. Second, we create and publicly release two new annotated datasets, PBL-HEK and PBL-N2a, providing much-needed benchmark resources for evaluating cross-dataset generalization performance of cell segmentation algorithms across diverse cell morphologies and imaging conditions. These datasets contain phase-contrast microscopy images of cell lines commonly used in biological research but captured with different microscopes than those in existing training datasets, facilitating a realistic assessment of model generalization.” in section 1.*

**4. LIVECell (5000 images) and Cytoplasm (600 images) are not that large datasets.**

*Thanks for this comment. We have removed several instances where we refer to these datasets as ‘large’ and we have added the following clarification to the paper “While 5000 images may not be considered large in the context of…”. In summary, while LIVECell and Cellpose Cytoplasm are not large datasets in the context of a standard computer vision training dataset, they are unprecedently large data sets for the field of annotated cell images. At the time of model training, these are the only relevant datasets available with regards to developing a generalist model. Given that within each image there are hundreds of segmented cells, even though LiveCell only has 5000 images, there are 1.7 individual cells which the model is being trained on. For reference, it can take up to 20 minutes to annotate a cell image by hand. The lack of training data has been a significant issue in this field. This is one of our primary reasons for using SAM, which is already pre-trained, and does not require as much data for fine tuning tasks.*

**5. Figures in general lack details. The network details should be also visible from the architecture figure.**

*Thank you for this suggestion. The network architecture is not novel and was published in the original Segment Anything Model publication, which we have cited in the paper. We have also included a reference figure of SAM from the original paper. The intention is that we can abstract away the internal details of the Segment Anything Model, emphasizing instead the finetuning and the overall segmentation algorithm. To this end, we have added a detailed diagram of the SAMCell pipeline, complete with a view of our training data preparation, finetuning method, and inference using the watershed algorithm. We have added the following sentence in section 2.5 as well: “We retain the original architecture of the Segment Anything Model (SAM) and abstract away its internal details for simplicity.”*

**6. In the Sliding Window Approach, what is the reasons of cropping out 256x256px patches from the input images, then upsample to 1024x1024px? Is it better than directly upsampling the input images to 1024x1024px ?**

*Thanks for this question, in our new ablation study on patch sizes in appendix A.3, we have clarified that our approach maintains SAM's original input/output dimensions (1024×1024 input, 256×256 output) to leverage the pretrained model architecture without modifications. We have also added a deeper discussion on this topic in section 2.5.2 “We chose this sliding window approach over directly upsampling…”. For this work, we chose not to change the model architecture, and instead implemented a sliding window to restitch the images after the model ran.*

**7. What is the reasons to stop on 40th epoch? What if you train more?**

*Thank you for the comment. Upon further reflection, we agreed with the need for more precise understanding of training and model convergence. During the re-trainings conducted during this revision, we adopted a different training protocol as outlined in section 2.7 (Training Protocol). “We trained using early stopping over a range of 35 to 100 epochs, with a patience of 7 epochs and a minimum improvement threshold of 0.0001. We save the model after every checkpoint and take the version with the lowest loss…”. We found that for most configurations, our models stopped training after 30-35 epochs because of this criterion, proving that the model training had converged and training more would have no significant advantage. This resulted in higher perfomance than previously seen, so we would like to humbly thank our reviewer for driving this change.*

**8. The paper need more ablation studies:**
**1. Different sized patches.**
**2. Different threshold values in the post-processing.**
**3. Segmenting examples of different shaped cells (dividing, c-shape etc)**
**4. How the segmentations changed with each (or every 10) epochs?**

*Thank you for this suggestion. Upon further reflection, we agreed with the need for more precise ablation studies and actually upon performing them, we were able to optimize our parameters even further. This resulted in higher perfomance than previously seen, so we would like to humbly thank our reviewer for driving this change.*

*To this end, we have conducted a deeper pretraining ablation study, as well as four other ablation studies, all in appendix A. While we had done a course check for these parameters during our initial model training, we did a finer sweep and found some interesting results that we have added to the paper. Some of these changes have positively impacted the accuracy of our models as well, which are reflected in the updated metrics values. We have added an ablation study about different patch sizes, model variants, different threshold values in post processing, as well as different datasets for training. We have added how the segmentation metrics changed every 5 epochs in the pretraining ablation study for both random initialization and pretrained weights, giving interesting results. (appendix A.4). We have also added examples from PBL HEK and PBL N2a, which contain cell morphologies found in real scenarios, segmented using cellpose cyto (our best performing baseline) and SAMCell to understand how they differ qualitatively (section 3.5).*

**9. Table 1 seems unfair since SAMCell should be finetuned, and the baselines are trained from the scratch (or were they finetuned also?).**

*Thank you for the note. We have added this sentence in the caption "The Stardist, Cellpose, and CALT-US models were trained only on LIVECell-train then tested on LIVECell-test (top) and separately trained only on Cyto-train, then tested on Cyto-test (bottom). SAMCell, inheriting SAM's pretraining, was fine-tuned only on LIVECell-train then tested on LIVECell-test (top) and separately fine-tuned only on Cyto-train and then tested on Cyto-test (bottom)." To clarify, for table 1, the three baselines were trained on livecell and cyto from scratch and samcell was fine tuned using the same datasets. We have hypothesized that the reason samcell is able to get high accuracy is because of its architecture, and also its pretraining. This is one of the core advantages of using SAM - since it already has a prior for object segmentation, we should be able to get higher accuracy.*

**10. For Table 2, it is still better to show the scores of the baselines trained on the LIVECELL dataset. I believe they all have pretrained weights, also.**

*Thank you for this suggestion, unfortunately this is beyond the scope of this study. While this is a good idea, we ran into errors trying to load some of these weights. Addressing this would require significant training time and computational resources. We will consider pursuing these baselines for future work.*

**11. It is more comprehensive if segmentation comparisons from baselines and two-SAMCell models are shown.**

*Thank you for this note. For table 1, the values at the top represent all four models trained on LIVECell-train and then tested on the LIVECell-test datasets, and the values at the bottom represent all four models trained on Cyto-train and then tested on Cyto-test. This is to understand how well these models perform on data similar to the data they have encountered during training. This clarification has been added to the table caption, For Table 2, we now compare 3 variants of samcell with 3 of our baselines on zero shot datasets to understand each model’s performance with images it has never seen before.*

**12. User interface seems nice. It is also better to consider creating a plugin for image viewers, such as Napari or Imagej.**

*Thank you for this suggestion. We agree that a napari plugin would enhance the user experience even further since they will be able to manually correct any errors. To this end, we are developing a fully functioning plugin for napari called samcell-napari which is currently available on the napari hub, and is still being optimized. However, this is outside the scope of this study so we elect to omit this from the paper.*

**"Overall, the paper is well-written. However, there are still some gaps that need to be addressed to reproduce the results, especially inside the figures. In my opinion, there are not enough ablation studies to support the hyperparameter settings and other decisions, and the comparisons with the baselines are insufficient to support the claims. In its current version, this paper is more suitable to be submitted to a conference (could be top-tier).**

*We appreciate the suggestions and advice. We have made substantial revisions in response to the thoughtful suggestions of the reviewers, in order to meet your approval for publication in this journal. Once again, we humbly thank the reviewers for their insightful feedback as we work to publish this article.*
